# Archaeal Mo-Containing Glyceraldehyde Oxidoreductase Isozymes Exhibit Diverse Substrate Specificities through Unique Subunit Assemblies

**DOI:** 10.1371/journal.pone.0147333

**Published:** 2016-01-25

**Authors:** Takayoshi Wakagi, Hiroshi Nishimasu, Masayuki Miyake, Shinya Fushinobu

**Affiliations:** 1 Department of Biotechnology, Graduate School of Agricultural and Life Sciences, The University of Tokyo, Yayoi, Bunkyo-ku, Tokyo, Japan; 2 Department of Biological Sciences, Graduate School of Science, The University of Tokyo, Yayoi, Bunkyo-ku, Tokyo, Japan; 3 PRESTO, JST, Yayoi, Bunkyo-ku, Tokyo, Japan; University of Queensland, AUSTRALIA

## Abstract

Archaea use glycolytic pathways distinct from those found in bacteria and eukaryotes, where unique enzymes catalyze each reaction step. In this study, we isolated three isozymes of glyceraldehyde oxidoreductase (GAOR1, GAOR2 and GAOR3) from the thermoacidophilic archaeon *Sulfolobus tokodaii*. GAOR1–3 belong to the xanthine oxidoreductase superfamily, and are composed of a molybdo-pyranopterin subunit (L), a flavin subunit (M), and an iron-sulfur subunit (S), forming an LMS hetero-trimer unit. We found that GAOR1 is a tetramer of the STK17810/STK17830/STK17820 hetero-trimer, GAOR2 is a dimer of the STK23390/STK05620/STK05610 hetero-trimer, and GAOR3 is the STK24840/STK05620/STK05610 hetero-trimer. GAOR1–3 exhibited diverse substrate specificities for their electron donors and acceptors, due to their different L-subunits, and probably participate in the non-phosphorylative Entner-Doudoroff glycolytic pathway. We determined the crystal structure of GAOR2, as the first three-dimensional structure of an archaeal molybdenum-containing hydroxylase, to obtain structural insights into their substrate specificities and subunit assemblies. The gene arrangement and the crystal structure suggested that the M/S-complex serves as a structural scaffold for the binding of the L-subunit, to construct the three enzymes with different specificities. Collectively, our findings illustrate a novel principle of a prokaryotic multicomponent isozyme system.

## Introduction

Molybdenum-containing hydroxylases are involved in diverse biological processes, and classified into the xanthine oxidoreductase (XOR), sulfite oxidase and DMSO reductase families [1–7]. The XOR family includes XOR (xanthine dehydrogenase, XDH, and xanthine oxidase, XO), carbon monoxide dehydrogenase (CODH), and aldehyde oxidoreductase (AOR). The XOR family belongs to the molybdo-pyranopterin (Mo-PPT) cofactor-containing enzyme superfamily. XORs have the mononucleotide form of the Mo-PPT cofactor, whereas the other XOR family members have molybdo-pyranopterin cytosine dinucleotide (Mo-PCD) at their active sites. The reaction mechanisms of the XOR family enzymes have been studied extensively [6]. The reaction starts with proton abstraction from the equatorial Mo-OH group by the conserved Glu residue, followed by hydride transfer from the substrate to the Mo = S group. During catalysis, two electrons are derived from the substrate hydroxylation, and are transferred via intrinsic cofactors (two [2Fe-2S] clusters and FAD) to the final electron acceptors, such as oxygen and NAD^+^.

**Fig 1:**
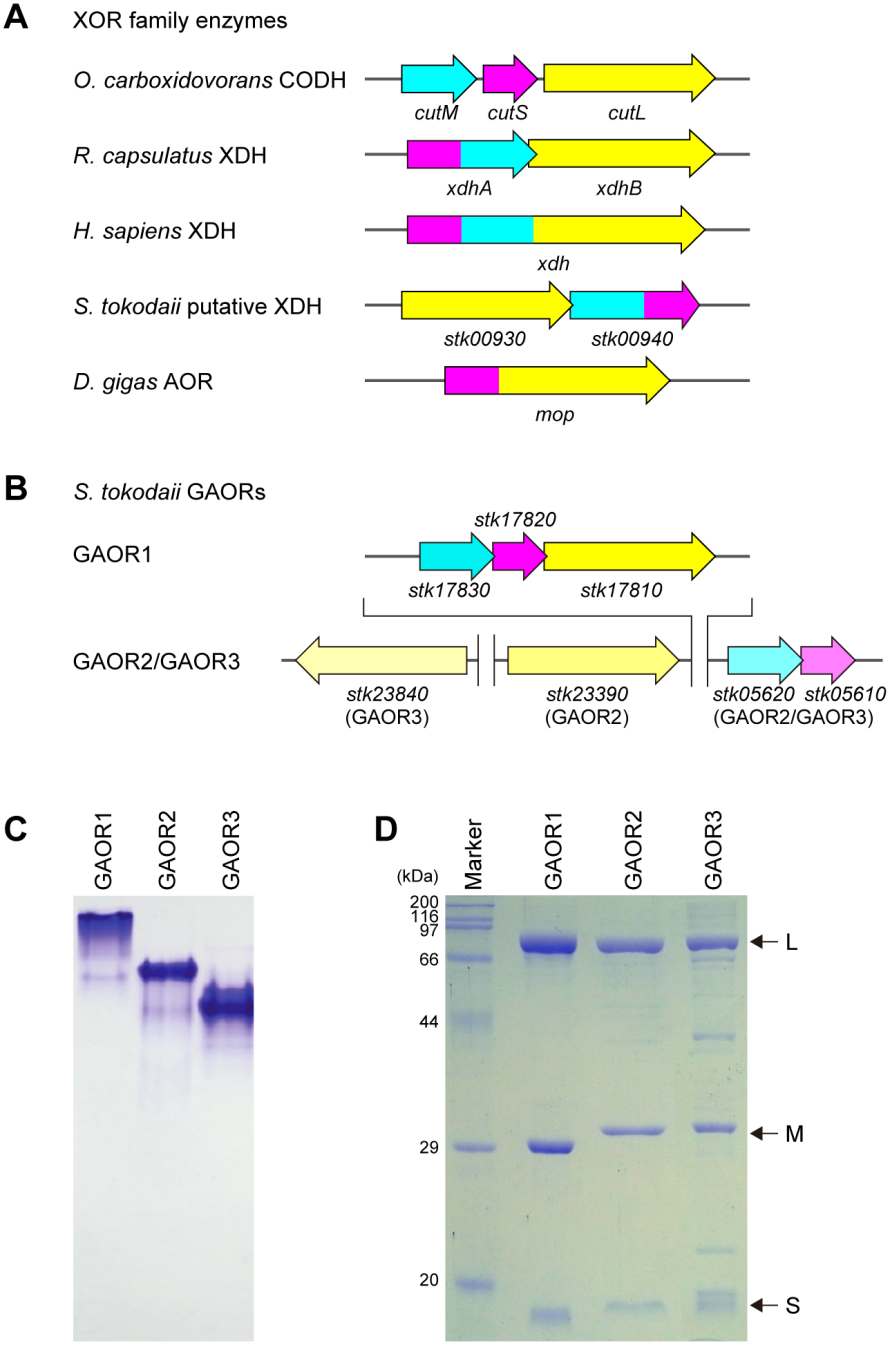
The XOR family enzymes and the *S*. *tokodaii* GAORs. (**A** and **B**) Gene arrangements of the XOR family enzymes (**A**) and the *S*. *tokodaii* GAORs (**B**). *O*. *carboxidovorans*, *Oligotropha carboxidovorans*; *R*. *capsulatus*, *Rhodobacter capsulatus*; *D*. *gigas*, *Desulfovibrio gigas*; CODH, CO dehydrogenase; XDH, xanthine dehydrogenase; AOR, aldehyde oxidase. In (**A**) and (**B**), the L-, M- and S-subunits are indicated by yellow, cyan and magenta arrows, respectively. (**C** and **D**) Non-denaturing PAGE (**C**) and SDS-denatured PAGE (**D**) of the purified GAOR proteins. In (**D**), the purity of GAOR3 was estimated to be 70%, using ImageJ.

The XOR family enzymes are found throughout all three domains of life [4,7,8] (Fig 1A). Most of the XOR family enzymes are composed of three fundamental polypeptide components, S (approximately 20 kDa, with iron-sulfur clusters), M (approximately 30 kDa, with FAD), and L (80–90 kDa, with Mo-PPT/Mo-PCD cofactor). Bacterial CODHs are dimers of hetero-trimers consisting of three separate subunits, M (32 kDa), S (18 kDa) and Mo-PCD-containing L-subunits (90–97 kDa). Bacterial XDHs are dimers of hetero-dimers composed of a 50 kDa XDHA subunit comprising an N-terminal iron-sulfur domain and a C-terminal FAD domain, and an 85 kDa Mo-PPT-containing XDHB subunit [9]. Mammalian XDHs are homodimers of a 140 kDa single polypeptide comprising S-, M- and L-domains, in this order. Some of the XOR family members lack the FAD-component; for example, AOR from *Desulfovibrio gigas* (DgAOR) and AOR from *Desulfovibrio desulfuricans* are homo-dimers composed of a 98-kDa polypeptide, comprising an N-terminal iron-sulfur domain and a C-terminal Mo-PCD domain.

Archaeal glycolysis is divergent from its eukaryotic and bacterial counterparts, and can be classified into the modified Embden-Meyerhof-Parnas pathway and the modified Entner-Doudoroff (mED) pathway, including the non-phosphorylative Entner-Doudoroff (npED) pathway [10,11] (S1 Fig). Previous studies indicated that glyceraldehyde (GA) dehydrogenases (GADHs) are responsible for the GA oxidation, in the npED pathways in the thermoacidophilic euryarchaeota *Picrophilus torridus* and *Thermoplasma acidophilum* [12]. GADHs are homo-dimers consisting of two 55 kDa subunits belonging to the aldehyde dehydrogenase superfamily. In contrast, the GA-oxidizing enzymes in the npED pathway have not been definitely identified. One candidate is a Mo-containing glyceraldehyde oxidoreductase (GAOR), which was first isolated from the thermoacidophilic archaeon, *Sulfolobus acidocaldarius* [13]. The enzyme (SaGAOR) oxidizes GA to glycerate [10,11] (S1 Fig). SaGAOR consists of three subunits, L (80.5 kDa), M (32 kDa), and S (19.5 kDa), which contain Mo-PPT, FAD, and an iron-sulfur cluster, respectively [13]. A Mo-containing bifunctional enzyme (GAOR1) was subsequently isolated from *Sulfolobus tokodaii* [14] (Fig 1B). GAOR1 catalyzes not only the oxidation of various aldehydes, but also the decarboxylation of aromatic 2-oxoacids, such as indolepyruvate [14]. The partial N-terminal sequences indicated that the L/M/S subunits of SaGAOR and GAOR1 are encoded by *saci2271*/*saci2269*/*saci2270* and *STK17810*/*STK17830*/*STK17820*, respectively. Saci2271, Saci2269 and Saci2270 share 68, 51 and 82% identities with STK17810, STK17830 and STK17820, respectively. A proteomic analysis indicated that *Sulfolobus solfataricus* also has a GAOR enzyme (Sso2639/Sso2636/Sso2637 for the L/M/S subunits) [15], although its function remains unknown. GAOR1 and SaGAOR can oxidize various aldehydes, including GA, thus suggesting that they may be responsible for the GA oxidation in the npED pathway. However, it remains elusive whether these GAORs are physiological GA-oxidizing enzymes, since the GA-oxidizing activities in archaeal cell extracts have not been fully investigated.

To identify the enzyme responsible for the GA oxidation in the npED pathway, we carefully isolated the GA-oxidizing activities in the cell-free extract of *S*. *tokodaii*, and obtained three purified enzymes (GAOR1, GAOR2 and GAOR3). Biochemical analyses revealed that GAOR1–3 showed different substrate specificities for various electron donors and acceptors, and suggested the involvement of GAOR2 in glycolytic GA oxidization. The subunit compositions of GAOR2 and GAOR3 indicated that the common set of the M/S complex binds to different L-subunits. Moreover, we solved the X-ray crystal structure of GAOR2, to obtain insights into the substrate specificities and the subunit assemblies of the three GAORs. Based on these findings, we propose a new principle of subunit assembly for the prokaryotic multicomponent isozymes.

## Materials and Methods

### Preparation of GAOR

*S*. *tokodaii* cells were cultured heterotrophically at pH 3 and 75°C, with vigorous aeration, collected by centrifugation at mid-log phase, and then stored at −80°C, as described [16]. The cells (36 g wet weight) were resuspended in 120 mL of 50 mM Tris-HCl, pH 8.0, and then disrupted by sonication using a Branson Sonifier (50% duty cycle, 1 s interval, for 10 min at 80 W). The cell-free extracts were supplemented with 0.1 mM phenylmethylsulfonylfluoride and a trace amount of DNase, incubated at room temperature for 1 h to digest the genomic DNA, and then centrifuged at 100,000 × g for 1 h. The supernatant was designated as the cell-free extract. The GAOR enzymes were purified at room temperature by a combination of chromatographies on DEAE cellulose DE52 (Whatman), Butyl Toyopearl 650M (Tosoh), Q-Sepharose (GE Healthcare), hydroxylapatite (BioRad) and Superdex 200 10/30 (GE Healthcare) columns. The standard buffers used were buffer I (20 mM Tris-HCl, pH 7.5, and 0.1 mM EDTA) and buffer II (20 mM Tris-HCl, pH 7.5, and 0.25 M NaCl).

A typical purification procedure is shown in S2A Fig. The cell-free extract was applied to a DEAE cellulose column (5.0 × 18 cm, 350 mL), and the proteins were eluted at a flow rate of 10 mL/min, with a linear gradient of NaCl from 0 to 0.4 M in buffer I in a total volume of 1,120 mL (S2B Fig). The enzyme activity was detected in a single peak at 0.15–0.2 M NaCl (fraction D). The active fraction (D) was supplemented with solid ammonium sulfate to a final concentration of 1.6 M, and then applied to a Butyl Toyopearl column (2.5 × 8 cm, 39 mL). The proteins were eluted at a flow rate of 3 mL/min, with a linear gradient of ammonium sulfate from 1.6 to 0 M in buffer I in a total volume of 360 mL, followed by 180 mL of buffer I (S2C Fig). The three peaks of the GA-oxidizing activity (DB1, DB2 and DB3) were separated. The three active fractions were desalted by ultrafiltration, and then applied to a Q-Sepharose column (1 × 10 cm, 7.8 mL). The proteins were eluted at a flow rate of 1 mL/min, with a linear gradient of NaCl from 0 to 0.25 M in buffer I in a total volume of 80 mL (S2D–S2F Fig). The active fractions (DB1Q, DB2Q and DB3Q) were desalted by ultrafiltration, and then applied to a hydroxyapatite column (1 × 5 cm, 3.9 mL) equilibrated in 5 mM sodium phosphate, pH 7.0. The proteins were eluted at a flow rate of 1 mL/min, with a linear gradient from 5 to 250 mM sodium phosphate buffer, pH 7.0, in a total volume of 60 mL. The active fractions were designated as DB1QH, DB2QH and DB3QH. These active fractions were concentrated by a Vivaspin turbo Ultra 15 filter (Sartorius). The samples (0.1 mL) were applied to a Superdex 200 10/30 gel filtration column equilibrated with buffer II, and then eluted at a flow rate of 0.5 mL/min. The active fractions were designated as DB2QHG (GAOR1), DB1QHG (GAOR2) and DB3QHG (GAOR3). A_280_ and A_400_ values were measured in a 1 mm light path, using a NanoDrop 3300 fluorospectrometer (Thermo Scientific).

### Chemicals

D,L-glyceraldehyde-3-phosphate was purchased from Santa Cruz Biotechnology, Inc. D-glyceraldehyde (GA) and other reagents were purchased from Sigma-Aldrich.

### Enzyme activity assay

During the purification steps, the GA-oxidizing activity of each fraction (5 μL) was measured at 70°C in a standard assay mixture. Standard assays were performed at 70°C, using GA and dichlorophenolindophenol (DCPIP) as the electron donor and acceptor, respectively, in a 400 μL reaction mixture containing 50 mM MOPS-NaOH, pH 7.0, 2 mM GA, 0.1 mM DCPIP and the sample protein. The reaction was started by the addition of the sample protein, and then the reduction of DCPIP was monitored by the decrease of the absorbance at 600 nm. One unit (U) of enzyme activity is defined as 1 μmol DCPIP reduced/min (ε_600_ = 21 mM^−1^ cm^−1^) [17]. Activity measurements using MV as an electron acceptor were performed in a 400 μL mixture, containing 50 mM MOPS-NaOH, pH 7.0, 2 mM GA, 2 mM MV and sample enzyme, in a 1.5-mL sealed cuvette containing Ar gas to purge the oxygen. The reaction was started by raising the temperature to 80°C, and the reduction of MV was monitored by the increase of the absorbance at 578 nm. One unit (U) of enzyme activity is defined as 1 μmol MV reduced/min (ε_578_ = 9.8 mM^−1^ cm^−1^) [18]. Ferredoxin (Fd) was purified from the cell-free extract, as described previously [19]. To examine the ability of Fd to accept electrons from GA, activity assays were performed at 70°C, using GA and Fd as the electron donor and acceptor, respectively, in a 400 μL reaction mixture containing 50 mM MOPS-NaOH, pH 7.0, 2 mM GA, 0.02 mM Fd and the sample protein. The reaction was started by the addition of the protein sample, and then the reduction of oxidized Fd was monitored by the decrease of the absorbance at 408 nm [19]. The enzyme kinetic data were obtained in triplicate experiments, and fitted to the Michaelis-Menten equation using KaleidaGraph (Hulinks). Enzyme turnover was calculated by assuming 1 catalytic site/mol LMS hetero-trimer; i.e., 131,744, 126,955 and 129,912 g of GAOR1, GAOR2 and GAOR3, respectively. One mg of GAOR1, GAOR2 and GAOR3 correspond to 7.59, 7.88 and 7.70 nmol of each LMS hetero-trimer, respectively.

### Protein amount and molecular weight estimation

Protein concentrations were determined by the bicinchoninic acid assay method (Pierce Chemical Co.), using bovine serum albumin as the standard. The molecular weights of GAORs were estimated by Superdex 200 10/30 gel filtration chromatography, using protein standards (BioRad): thyroglobulin (670 kDa), γ-globulin (158 kDa), ovalbumin (44 kDa), myoglobin (17 kDa) and vitamin B_12_ (1.35 kDa). The molecular weights of SDS-denatured GAOR subunits were estimated by 14% polyacrylamide gel electrophoresis (PAGE), using protein standards (BioRad). The sample proteins were denatured with 2% SDS and 2% 2-mercaptoethanol at 100°C for 10 min, and the proteins were visualized by Coomassie Brilliant Blue staining [20]. Native PAGE was performed in the same electrophoresis system as above, except that SDS was omitted from every component and a 7% polyacrylamide gel was used. The theoretical isoelectric points (pIs) were calculated using the ExPASy web site (http://web.expasy.org/compute_pi/).

### N-terminal amino-acid sequence determination

The purified GAORs were fractionated by SDS-PAGE, and blotted onto a polyvinylidene fluoride membrane. The N-terminal amino-acid sequences of each subunit were determined using an Applied Biosystems Model 370A automated gas phase protein sequencer (Applied Biosystems).

### Metal content determination

The metal content was determined using an 820-MS inductivity coupled plasma (ICP) mass spectrometer (Varian). The sample proteins (3.9–8.6 mg/mL) were hydrolyzed, oxidized in 0.1 N nitric acid [14], and then diluted 500-fold. Serial dilutions of a standard solution containing Mo, Fe, Cu, Se, or W, each at a final concentration of 0.5 and 1 ppb, were also analyzed.

### Crystallography

Crystallization was performed at 25°C, using the sitting drop vapor diffusion method. Crystals were obtained by mixing 0.5 μL of protein solution, consisting of 8 mg/mL GAOR2, 5 mM Tris-HCl, pH 8.0, 2 mM indoleacetate and 0.5% dimethyl sulfoxide, and 1 μL of reservoir solution, consisting of 0.1 M Na-acetate, pH 4.5, 0.2 M CH_3_COONH_4_ and 15% (w/v) PEG 4,000. The X-ray diffraction data set was collected at the BL40B2 station at SPring-8 (Hyogo, Japan). The crystal was cryoprotected in the reservoir solution supplemented with 20% PEG400, and was flash-cooled at 100 K in a stream of nitrogen gas. The diffraction data were processed with HKL2000 [21]. Molecular replacement was performed with MOLREP [22]. Automated model building, manual model rebuilding and structural refinement were performed using ARP/wARP [23], Coot [24] and Refmac [25], respectively. The final model contains one hetero-trimer (LMS), one set of cofactors (one Mo-PCD, one FAD, and two [2Fe-2S] clusters), one acetate, six low-molecular weight PEGs (four C_4_H_10_O_3_, one C_8_H_18_O_5_ and one C_10_H_22_O_6_) and 642 waters. Although no ligand was observed in the active center, the addition of indoleacetate facilitated crystal growth. Molecular graphic images were prepared using CueMol (http://www.cuemol.org) and PyMol (Schrödinger, LLC).

## Results

### Purification of GAOR1–3

To identify the GA-oxidizing enzyme in the glycolytic pathway in *S*. *tokodaii*, we purified the GA-oxidizing activities from the cell-free extract, using DCPIP as an electron acceptor. After several column chromatography steps, we isolated three GA-oxidizing enzymes (designated as GAOR1, GAOR2 and GAOR3) from the *S*. *tokodaii* cell extracts. The results of a typical purification are summarized in Table 1. The three purified GAORs were dark red in color, and migrated as faint red bands on native PAGE (further stained with Coomassie Brilliant Blue in Fig 1C), suggesting the presence of the Mo cofactor, FAD and iron-sulfur clusters. The SDS-PAGE analysis indicated that all three of the GAORs contain three major bands, corresponding to the L, M and S subunits (Fig 1D). The purity of GAOR3 was estimated to be approximately 70% (Fig 1D). GAOR3 was relatively unstable, and lost half of its activity within a month at 4°C. The native PAGE analysis revealed the different mobilities of the three GAORs (GAOR3 > GAOR2 > GAOR1), which mainly depend on their molecular sizes, since their pIs are calculated to be lower than the pH during native PAGE (pH 8.9) (the pIs of GAOR1, GAOR2 and GAOR3 are 6.94, 6.29 and 6.16, respectively). These results suggested the differences in the molecular weights of the three GAORs (GAOR1 > GAOR2 > GAOR3).

**Table 1:** Summary of a typical purification.

Step	Fraction	Activity	Protein	Specific activity	Purification	Figure	Remarks
		(U)	(mg)	(U/mg)	(fold)		
Cell-free extract		199	3,620	0.055	1	S2A	
DE52	D	257	1,176	0.219	3.97	S2B	
Butyl-650M	DB1	51.8	119	0.435	7.91	S2C	
	DB2	25.9	179	0.145	2.60	S2C	
	DB3	39.8	126	0.315	5.74	S2C	
Q-Sepharose	DB1Q	34.3	17.4	1.97	35.8	S2D	
	DB2Q	8.30	21.8	0.38	6.92	S2E	
	DB3Q	15.6	25.0	0.624	11.3	S2F	
Hydroxyapatite	DB1QH	13.3	7.43	1.79	32.5	-	
	DB2QH	2.25	5.57	0.404	7.34	-	
	DB3QH	16.6	7.16	2.32	42.2	-	
Superdex 200	DB1QHG	4.39	1.59	2.76	50.2	S2G	GAOR2
	DB2QHG	1.04	0.76	1.36	24.7	S2G	GAOR1
	DB3QHG	2.27	1.29	1.76	32.0	S2G	GAOR3

The N-terminal amino-acid sequence analysis revealed that the genes encoding the L/M/S subunits of GAOR1, GAOR2 and GAOR3 are *stk17810*/*stk17830*/*stk17820*, *stk23390*/*stk05620*/*stk05610* and *stk24840*/*stk05620*/*stk05610*, respectively (Table 2). Unexpectedly, GAOR2 and GAOR3 commonly share the M (STK05620) and S (STK05610) subunits. The apparent molecular weights of the L-, M- and S-subunits are consistent with the theoretical molecular weights (Fig 1D). According to the NCBI genome database, STK17810, STK17830 and STK17820 have been annotated as the CODH L-, S- and M-subunits, respectively. STK23390 and STK24840 have been annotated as the CODH L-subunit and AOR, respectively. STK05620 and STK05610 have been annotated as the CODH M-subunit and a “dehydrogenase”, respectively.

**Table 2:** Characterization of the three GAORs.

	GAOR1	GAOR2	GAOR3 ^a^
Molecular mass ^b^	371 kDa (526,976 Da)	200 kDa (253,910 Da)	85 kDa (129,912 Da)
Gene			
L	*stk17810* (744 aa)	*stk23390* (706 aa)	*stk24840* (730 aa)
M	*stk17830* (283 aa)	*stk05620* (278 aa)	*stk05620* (278 aa)
S	*stk17820* (167 aa)	*stk05610* (168 aa)	*stk05610* (168 aa)
Subunit molecular mass ^c^			
L	83 kDa (81,947 Da)	81 kDa (77,626 Da)	82 kDa (80,583 Da)
M	29 kDa (31,255 Da)	30 kDa (30,710 Da)	30 kDa (30,710 Da)
S	20 kDa (18,542 Da)	21 kDa (18,619 Da)	21 kDa (18,619 Da)
Subunit composition	(LMS)_4_	(LMS)_2_	LMS
Metal content (nmol/mg protein)			
Mo	5.53	4.46	2.9
Fe	26.2	18.6	10.5
Cu	0.30	0.56	0.76
Se	0	0.026	0.07
W	0	0	0
Metal ratio (mol/mol)			
Fe/Mo	4.6	4.2	3.7
Cu/Mo	0.05	0.13	0.26
Enzyme activity ^d^			
Electron acceptor			
Electron donor	*k*_cat_ (sec^−1^) / *K*_m_ (mM)	*k*_cat_ (sec^−1^) / *K*_m_ (mM)	*k*_cat_ (sec^−1^) / *K*_m_ (mM) ^a^
DCPIP (0.1 mM)			
GA	3.78 ± 0.18 / 0.138 ±0.020	50.5 ± 0.05 / 0.030 ± 0.009	3.58 ± 0.11 / 0.011 ± 0.00
MV (2 mM)			
GA	5.23 ± 0.13 / 0.63 ±0.06	11.2 ± 0.5 / 0.091 ± 0.014	0.72 ± 0.03 / 0.018 ± 0.002
GAP	0.98 ± 0.08 / 0.012 ±0.006	0.65 ± 0.01 / 0.085 ± 0.005	0.43 ± 0.03 / 0.0035 ± 0.0011
Acetaldehyde	3.23 ± 0.28 / 0.24 ±0.15	3.38 ± 0.02 / 1.33 ± 0.02	ND ^e^
Indoleacetaldehyde	3.46 ± 0.20 / 0.113 0.016	2.77 ± 0.22 / 0.092 ± 0.017	3.58 ± 2.49 / 0.37 ± 0.39
Indolepyruvate	11 ± 0.02 / 1.86 ± 0.62	0	0
Phenylpyruvate	1.0 ± 0.33 / 7.0 ± 4.0	0	0
NAD^+^ (2 mM)			
GA	0	0	0
NADP^+^ (2 mM)			
GA	0	0	0
Ferredoxin (0.02 mM)			
GA	0	0	0

^a^ Enzyme purity was approximately 70%, as deduced from the staining intensity of the protein bands on SDS-PAGE.

^b^ Estimation from gel filtration. Values in parentheses are theoretical values derived from the genome database (http://www.bio.nite.go.jp/dogan).

^c^ Estimation from SDS-PAGE.

^d^ Reactions using DCPIP/NAD(P)^+^ and MV were performed at 70°C and 80°C, respectively.

^e^ Not determined.

### Gene arrangement of GAOR1–3

The genes encoding the M/S-subunits, *stk05620*/*stk05610* and *stk17830*/*stk17820*, are located adjacently (overlapped) in this order for GAOR1–3 (Fig 1B). In GAOR1, the M/S-subunit gene loci precede the L-subunit gene locus, as observed in *Oligotropha carboxidovorans* CODH (OcCODH) [26]. In contrast, in GAOR2 and GAOR3, the L-subunit gene is located far from the M/S-subunit genes. The *S*. *tokodaii* genome has the genes *stk00930* and *stk00940*, which encode a putative xanthine oxidoreductase. The gene *stk00930* encodes the L-subunit, whereas the gene *stk00940* encodes the N-terminal flavin-binding M domain and the C-terminal iron-sulfur-binding domain. However, the functions of the *stk00930*/*stk00940* gene products remain unknown.

### Absorption spectra, cofactors and subunit compositions

Consistent with the fact that GAOR1–3 share sequence similarity with the XOR family enzymes, they showed ultraviolet/visible absorption maxima at 276–279 nm, 335–341 nm, 427–439 nm and 465 nm (shoulder), which are characteristic of the Mo cofactor, flavin and the iron-sulfur cluster, respectively (S3 Fig) [13,27,28]. Indeed, ICP-MS analyses revealed that the three GAORs contained Fe and Mo at a constant ratio of 3.7–4.6 (Table 2). A molar ratio of 4 for Fe/Mo is typical in the XOR family enzymes, which contain one Mo cofactor and two [2Fe-2S] clusters. The Mo content of GAOR3 was low (Table 2), probably due to the presence of impurities.

The gel filtration chromatography results suggested that the observed molecular masses of GAOR1, GAOR2 and GAOR3 are approximately 372 kDa, 200 kDa and 85.1 kDa, respectively (S2G Fig). The sums of the theoretical molecular weights of the three subunits LMS of GAOR1, GAOR2 and GAOR3 are 131 kDa, 127 kDa and 130 kDa, respectively. The molecular weights of oligomeric proteins from hyperthermophiles estimated by gel filtration chromatography are sometimes smaller than the theoretical ones, due to the compact assembly of their quaternary structures [19]. For example, the inorganic pyrophosphatase from *S*. *acidocaldarius* forms a homo-hexamer of 21 kDa subunits (PDB ID: 1QEZ), but the molecular weight was estimated by gel filtration to be 85 kDa [29]. We thus concluded that GAOR3 is likely to be an LMS hetero-trimer. Taken together, these observations indicate that GAOR1 is a tetramer of hetero-trimers, (LMS)_4_, where L = STK17810, M = STK17830, S = STK17820; GAOR2 is a dimer of hetero-trimers, (LMS)_2_, where L = STK23390, M = STK05620, S = STK05610; and GAOR3 is a monomer of hetero-trimer, LMS, where L = STK24840, M = STK05620, S = STK05610. GAOR2 and GAOR3 share the same set of the M/S-subunits (STK05620/STK05610), which assemble with the different L-subunits (STK23390 and STK24840).

### Substrate specificities

We examined the substrate specificities of GAOR1–3, using various combinations of electron donors and acceptors (Table 2). When DCPIP was used as an electron acceptor, the *k*_cat_/*K*_m_ values for the GA oxidation were 27, 1,680 and 325 s^−1^·mM^−1^ for GAOR1, GAOR2 and GAOR3, respectively. When MV was used as an electron acceptor, the *k*_cat_/*K*_m_ values for GA were 27, 123 and 40 s^−1^·mM^−1^ for GAOR1, GAOR2 and GAOR3, respectively. These results suggested that GAOR2 oxidizes GA more efficiently than GAOR1 and GAOR3. Moreover, the *k*_cat_/*K*_m_ values for various electron donors indicated that GAOR1, GAOR2 and GAOR3 prefer acetaldehyde/GAP, GA/indoleacetaldehyde and GAP/GA as substrates, respectively (Table 2). In contrast to GAOR2 and GAOR3, GAOR1 displayed low catalytic efficiency for GA-dependent reactions, and can use aromatic 2-oxoacids, such as indolepyruvate and phenylpyruvate, as an electron donor (Table 2), consistent with a previous study indicating that GAOR1 may catalyze the decarboxylation of aromatic 2-oxoacids to yield the corresponding aldehydes [14]. The different substrate specificities of GAOR1–3 are likely to be due to the structural differences in their Mo-PCD-containing L-subunits that accept an electron donor and their FAD-containing M-subunits that release an electron to an external acceptor.

The *k*_cat_/*K*_m_ values of GAOR1 and GAOR2 for GAP (7.6–8.3 s^−1^·mM^−1^) were much smaller than those of the non-phosphorylating glyceraldehyde-3-phosphate dehydrogenase (GAPN) (96.6 s^−1^·mM^−1^) and the phosphorylating glyceraldehyde-3-phosphate dehydrogenase (GAPDH) (48.4 s^−1^·mM^−1^) from the same organism [30]. GAPN and GAPDH are involved in the “semi-phosphorylated branch” of the mED pathway of *Sulfolobales* (S1 Fig). These results suggested that GAOR1 and GAOR2 are not the primary enzymes responsible for the oxidation of GAP into 3-phosphoglycerate [11]. As neither NAD^+^, NADP^+^ nor Zn-containing ferredoxin [19] were available as an electron acceptor for GAOR1–3, the physiological electron acceptor for the GAORs remains unknown. A previous studies suggested that the GAOR1 enzymes are involved in the glycolytic pathways in *S*. *solfataricus* and *S*. *acidocaldarius* [13,15]. In contrast, our kinetic analyses suggest that GAOR2 is most likely to be responsible for the GA oxidation *in vivo*, since the catalytic efficiency and the protein amount *in vivo* seem higher than those of GAOR1 and GAOR3.

### Crystal structure of GAOR2

**Fig 2:**
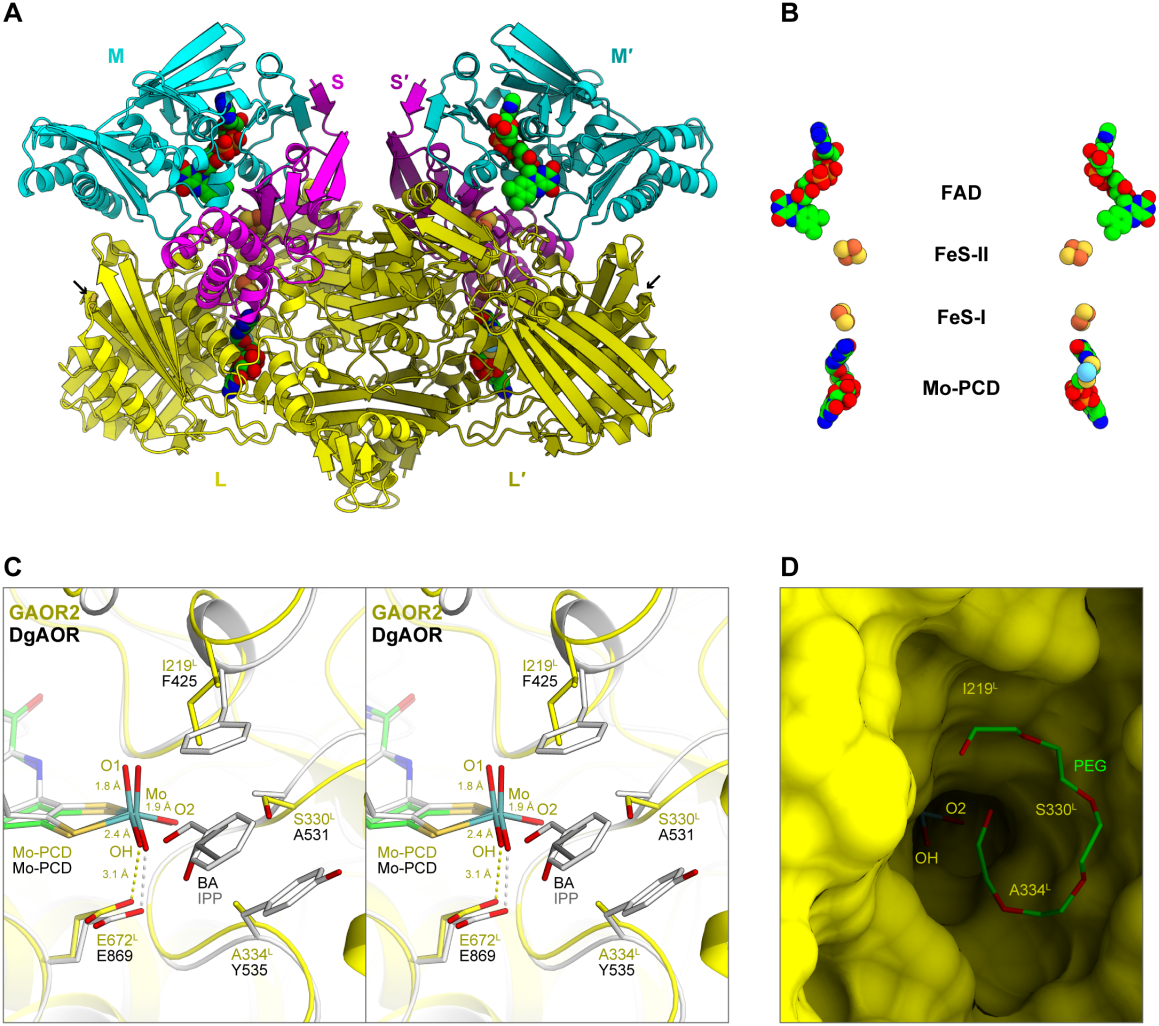
Crystal structure of GAOR2. (**A**) Overall structure of GAOR2. The L-, M- and S-subunits are colored yellow, cyan and magenta, respectively. The other hetero-trimer is indicated as L′, M′ and S′. The carbon atoms of the cofactors are colored green. Arrows indicate the disulfide bond between Cys405^L^ and Cys689^L^. (**B**) Arrangement of cofactors. (**C**) Catalytic center. GAOR2, isopropanol (IPP)-bound DgAOR (PDB ID: 1VLB, dark gray) and benzaldehyde (BA)-bound DgAOR (PDB ID: 4US8, gray) were superimposed. Since the IPP- and BA-bound DgAOR structures are essentially identical, the DgAOR structure in the IPP complex is omitted for clarity. The O1, O2, and OH indicate the apical oxo ligand, the basal oxo ligand, and a Mo-coordinating water molecule, respectively. (**D**) Molecular surface of the GAOR2 active site.

To understand the structural basis for the subunit assemblies and the substrate specificities of the GAORs, we solved the crystal structure of GAOR2 at 2.2 Å resolution by molecular replacement, using the structure of *Hydrogenophaga pseudoflava* CODH (HpCODH, PDB ID: 1FFU, sequence identity = 32/41/65% for the L/M/S subunits) [31] as the search model (Table 3). The electron densities of all of the cofactors are clearly observed (S4A–S4D Fig). The asymmetric unit contains one hetero-trimer (LMS complex), one Mo-PCD (L-subunit), one FAD (M-subunit), two [2Fe-2S] clusters (designated as I and II) (S-subunit), and solvent molecules. The two hetero-trimers are related by crystallographic 2-fold symmetry to form a functional (LMS)_2_ dimer of GAOR2, where the L-subunits mediate the dimerization (Fig 2A). GAOR2 has a butterfly-shaped, hetero-hexameric structure with a molecular size of approximately 140×65×90 Å, as observed for other XOR family enzymes (Fig 2A). Cys405^L^ and Cys689^L^ (superscripts L, M and S after the residue number indicate the subunit) form a disulfide bridge (Fig 2A). The other L-subunit proteins (STK17810 and STK24840) lack Cys residues. Structural similarity searches using the PDBeFold server [32] indicated that the M- and S-subunits are most similar to HpCODH (PDB ID: 1FFU, Q score = 0.78, root mean square deviation (RMSD) = 1.39 Å, and number of aligned Cα (Nalign) = 273 for M, and Q score = 0.91, RMSD = 0.62 Å, and Nalign = 154 for S), whereas the L-subunit is most similar to bovine XOR (PDB ID: 3B9J, Q score = 0.59, RMSD = 1.78 Å, and Nalign = 644).

**Table 3:** Data collection and refinement statistics.

PDB Accession code	4ZOH
**Data collection**	
Space group	*P*6_5_22
Cell dimensions (Å)	*a* = *b* = 143.4, *c* = 253.5
Resolution (Å)	50.0–2.20 (2.28–2.20)
Total reflections	885,394
Unique reflections	73,074 (7,170)
Completeness (%)	100 (100)
Redundancy	12.1 (12.3)
Mean *I*/*σ*(*I*)	36.7 (6.9)
*R*_sym_ (%)	7.8 (39.4)
**Refinement**	
Resolution (Å)	41.40–2.20
No. of reflections	69,288
*R*-factor / *R*_free_ (%)	17.6 / 24.2
No. of atoms	9,598
Protein atoms (A/B/C chains)	5,452 / 2,122 / 1,236
Cofactors (Mo-PCD / [2Fe-2S] / FAD)	48 / 8 / 53
Other ligands (PEGs and acetate)	61
Water molecules	618
Average *B*-factor (Å^2^)	
Protein atoms (A/B/C chains)	30.7 / 37.9 / 30.1
Cofactors (Mo-PCD / [2Fe-2S] / FAD)	25.4 / 22.1 / 27.9
Root mean square deviations from ideal values	
Bond lengths (Å)	0.020
Bond angles (°)	2.1
Ramachandran plot (%)	
Favored	96.4
Allowed	3.1
Disallowed	0.5

Values in parentheses are for the highest resolution shell.

### Cofactors and the active site

The Mo-PCD, [2Fe-2S] I, [2Fe-2S] II and FAD are located almost linearly, with distances of 13.4, 11.5 and 7.0 Å, respectively **(**Fig 2B). The Mo-PCD and FAD cofactors are recognized by many residues in the L- and M-subunits, respectively (S5A and S5B Fig). The two [2Fe-2S] clusters are coordinated by Cys residues in the S-subunit (S5B and S5C Fig). The ligand structure of the Mo atom is similar to that of the oxidized form of DgAOR (Fig 2C) [33]. In the GAOR2 structure, the Mo atom is penta-coordinated by two equatorial *cis* dithiolene sulfurs and three oxygen ligands: an apical oxo ligand (O1, 1.8 Å), a basal oxo ligand (O2, 1.9 Å), and a water molecule (OH, 2.4 Å). In the DgAOR structure (PDB ID: 1VLB), an isopropanol (inhibitor) molecule is bound to the active site [33]. In addition, the recently reported crystal structure of DgAOR in complex with benzaldehyde (PDB ID: 4US8) revealed that the aromatic ring of the benzaldehyde is sandwiched between the side chains of Phe425 and Tyr535 in the active site [34] (Fig 2C). Phe425 and Tyr535 of DgAOR are equivalent to Ile219^L^ and Ala334^L^ of GAOR2, respectively. Ala531 of DgAOR is also replaced with the hydrophilic Ser330^L^ in GAOR2. These structural differences can explain why GAOR2 efficiently oxidizes hydrophilic glyceraldehyde, whereas DgAOR prefers aromatic aldehydes. Although the present GAOR2 structure lacks a substrate, a polyethylene glycol molecule (pentaethylene glycol, C_10_H_22_O_6_) derived from the cyoprotectant solution is bound within a pocket formed by several residues in the L-subunit (Tyr190^L^, Ile219^L^, Asp292^L^, Asp293^L^, Gly296^L^, Asn297^L^, Ser330^L^, Arg336^L^, Phe417^L^ and Gly483^L^) (Fig 2D and S5C Fig). In the GAOR2 structure, Glu672^L^, a conserved catalytic Glu residue, is located at a position similar to those in other XOR family hydroxylases, such as DgAOR (Glu869) [35] (Fig 2C) and bovine XOR (Glu1261) [36], suggesting that the catalytic mechanism of GAOR2 is similar to those of other XOR family enzymes [37]. The electron flow during the GAOR2 reaction is predicted as follows: electrons are depleted from the substrate aldehyde to Mo-PCD, and are then transferred via [2Fe-2S] I, [2Fe-2S] II and FAD to external electron acceptors, such as DCPIP and MV (Fig 2B).

### Subunit assembly

**Fig 3:**
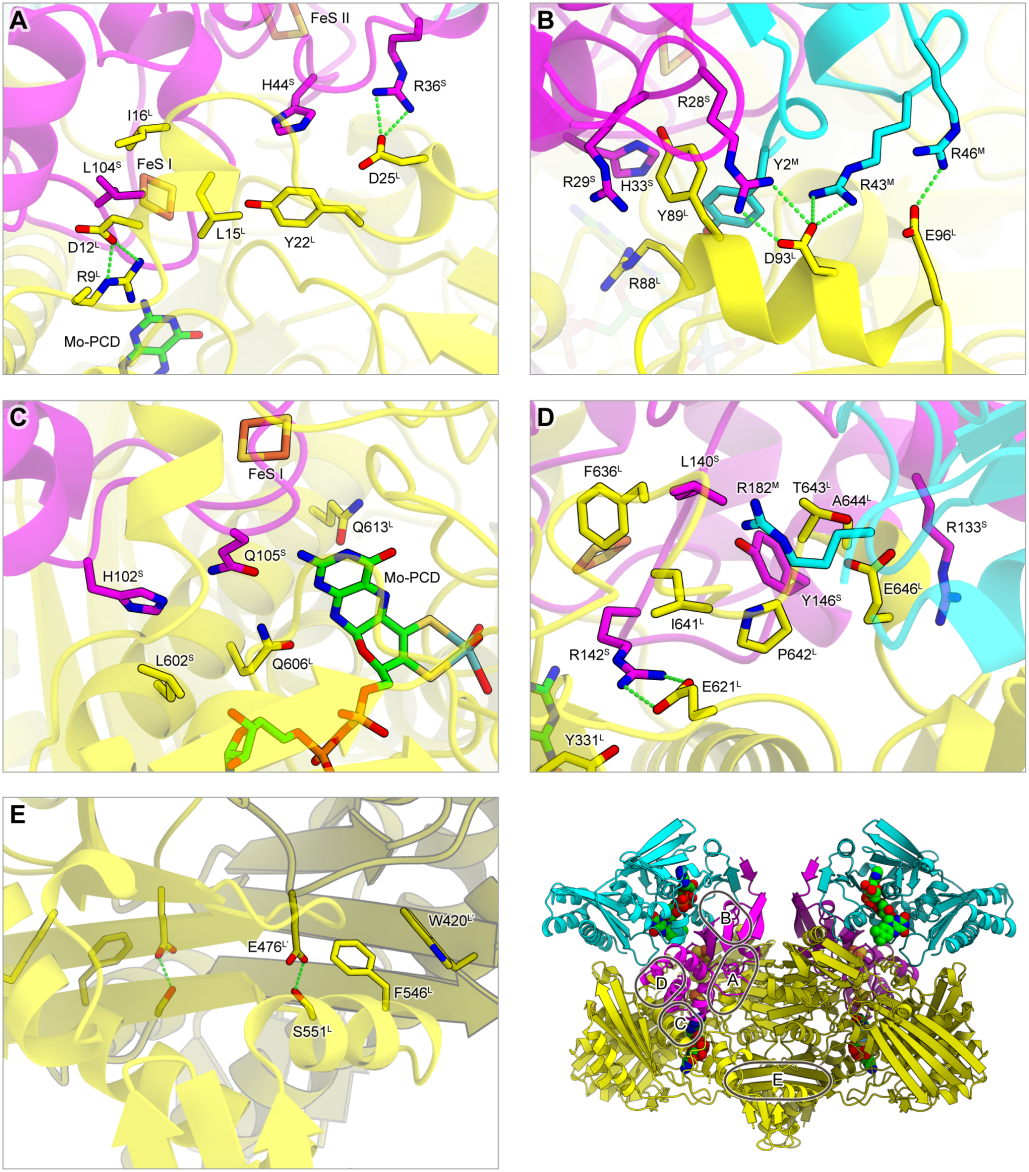
Inter-subunit interactions in GAOR2. (**A**–**E**) Major interfaces between the L-subunit and the M/S complex (**A**–**D**), and between the L- and L′-subunits (**E**).

The crystal structure and the sequence comparison provided mechanistic insights into the subunit assembly of the GAORs (Fig 2 and S6 Fig). The inter-subunit interactions in GAOR2 are summarized in S1 Table. As compared to the molecular surfaces of the subunits (26,200 Å^2^, 13,200 Å^2^ and 9,060 Å^2^ for L-, M- and S-subunits, respectively), the interface areas of L–S and L–L′ (dimer-related L-subunit) are relatively wide (>2,000 Å^2^), whereas those of M–S and L–M are relatively narrow (<1,600 Å^2^). There are no interactions between the other combinations of subunits. In GAOR2, the M–S and L–M interactions appear relatively weak, as in the OcCODH (LMS)_2_ dimer, in which its M-subunit can be removed with SDS and then reconstituted [38]. There are four major L–M/S interfaces (Fig 3A–3D), and the key residues at the interface are highly conserved in the L-subunits of GAOR2 (STK23390) and GAOR3 (STK24840) (S6 Fig), consistent with the compatibility of the M/S-subunits between GAOR2 and GAOR3.

Our biochemical and structural data showed that STK23390 (L of GAOR2) forms a homo-dimer. In contrast, STK24840 (L of GAOR3) is likely to be a monomer. Consistent with the differences in their subunit assemblies, the residues at the L–L′ interface of GAOR2 (STK23390) (Fig 3E) are not conserved in GAOR3 (STK24840) (S6 Fig). GAOR3 (STK24840) has a 9-residue insertion (Thr563^L^–Arg571^L^) in the region equivalent to the L–L′ interface (Phe546^L^–Asp553^L^) of GAOR2 (ST23390). In contrast to GAOR2 and GAOR3, GAOR1 forms a tetramer of hetero-trimers. GAOR1 (ST17810) has a unique 17-residue insertion (Ala70^L^–Arg96^L^) in the region equivalent to the solvent-exposed surface of GAOR2 (STK23390) (S6 Fig). These structural differences may be related to the distinct oligomeric states of the three GAORs, although further studies will be required to elucidate the underlying mechanism.

## Discussion

In this study, we purified three GAOR enzymes belonging to the XOR family, from the thermoacidophilic archaeon *S*. *tokodaii*. The GAOR1 enzymes from *S*. *acidocaldarius* (SaGAOR) [13] and *S*. *tokodaii* (previously referred to as indolepyruvate:MV oxidoreductase) [14] were previously characterized. These enzymes exhibit GA-oxidizing activity and are relatively abundant in the cytosol, suggesting their involvement in the GA oxidation in the npED pathway [15]. However, our biochemical data showed that in *S*. *tokodaii*, GAOR2 is more specific to GA, as compared with GAOR1, and indicated that GAOR2 is responsible for the GA oxidation in the npED glycolytic pathway in *Sulfolobales*.

In contrast to the mED pathway, a tungsto-bispyranopterin (W-bisPPT)-containing glyceraldehyde-3-phosphate:ferredoxin oxidoreductase is responsible for the oxidation of glyceraldehyde-3-phosphate into 3-phosphoglycerate, in the modified Embden-Meyerhof-Parnas glycolytic pathway of the anaerobic hyperthermophilic archaea [39]. The crystal structure of glyceraldehyde-3-phosphate:ferredoxin oxidoreductase from *Pyrococcus furiosus* (PfAFOR) has long been the sole three-dimensional structure of a metal-pyranopterin-containing enzyme from Archaea [40]. PfAFOR belongs to the W-bisPPT-containing enzyme family, and possesses the W-bisPPT cofactor consisting of a tungsten atom and two pyranopterin molecules [2]. The present crystal structure revealed that GAOR2 belongs to the Mo-PTT-containing XOR family and possesses a Mo-PCD. It is interesting that the Mo-PPT and W-bis-PPT oxidoreductases play important roles in the two distinct glycolytic pathways in Archaea.

A structural comparison of the L-subunit of GAOR2 (STK23390) with the homology models of GAOR1 (STK17810) and GAOR3 (STK24840) can partly explain their distinct substrate specificities (electron donor). In the homology model of STK24840, the substrate-binding pocket is narrower than that of STK23390, due to the replacement of STK23390 Ser330^L^ with STK24840 Gln338^L^ (S7A and S7B Fig). This is consistent with the result that GAOR3 exhibits less affinity to a relatively large substrate, indoleacetoaldehyde, than GAOR1 and GAOR2 (Table 2). In contrast, in the homology model of STK17810, the substrate-binding pocket is larger than that of STK23390, due to the replacement of STK23390 Ile219^L^ by STK17810 Val250^L^ (S7C Fig). This is consistent with the result that GAOR1 prefers larger substrates, as compared with GAOR2. It remains unclear why only GAOR1 can catalyze the decarboxylation reaction of 2-oxoacid substrates, indolepyruvate and phenylpyruvate. Together, our structural observations indicated that the L-subunits of the GAORs function in the definition of their substrate specificities.

**Fig 4:**
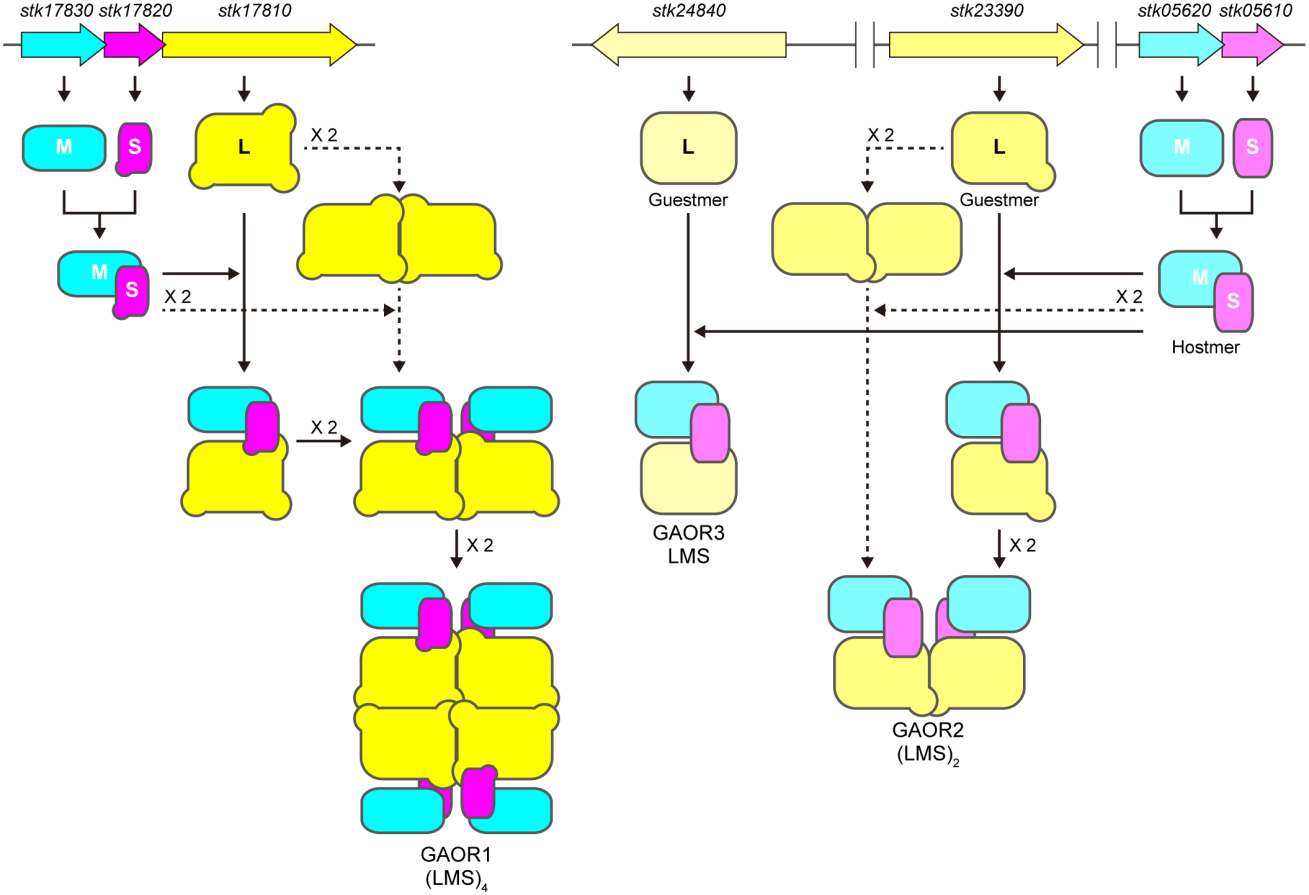
Possible assembly pathway of the three GAORs. Various pathways may be possible for L-subunit dimerization, with the simplest and alternative pathways shown by bold and dotted arrows, respectively.

The genes encoding the subunits of GAOR2 and GAOR3 are not clustered (Fig 1B), suggesting that their assembly pathways are different from that of GAOR1 (Fig 4). GAOR2 and GAOR3 contain the M/S (STK05610/STK05620) complex as a common component that recruits different L-subunits (STK23390 or STK24840). While the genes encoding the L-subunits (*stk23390* for GAOR2 and *stk24840* for GAOR3) are located at distant loci from *stk05610*/*stk05620*, the two genes *stk05610* (M subunit) and *stk05620* (S subunit) are adjacent to each other. The *stk05610* and *stk05620* genes are preceded by a promotor-like sequence, suggesting that they constitute an operon. These observations imply that the M/S-subunits (STK05610/STK05620) may assemble into the M/S hetero-dimer, which then interacts with a different L-subunit, either STK23390 (GAOR2) or STK24840 (GAOR3). In contrast to GAOR2 and GAOR3, GAOR1 is encoded by three successive genes, *stk17830* (M-subunit), *stk17820* (S-subunit) and *stk17810* (L-subunit). In GAOR1, the genes encoding the M/S-subunits also precede the genes encoding the L-subunit, thereby suggesting that in GAOR1, the M/S complex formation also occurs prior to the L-subunit association. These notions are supported by a recent study showing that bacterial gene organization into operons contributes to the effective assembly of protein complexes [41].

The exchange of subunit counterparts is observed in some eukaryotic hetero-oligomeric proteins, such as human hemoglobin and the G-protein heterotrimer complex. In the case of human hemoglobin, the α-subunit binds to the β- and γ-subunits in the periods before and after birth, respectively. The transition from α_2_γ_2_ to α_2_β_2_ hemoglobin after the birth of the fetus is quite reasonable, given their different oxygen saturation modes [42]. The human globin genes encoding the β- and γ-subunits are clustered on the same chromosome, while the gene encoding α-globin is located on a different chromosome. The signal transducing G-proteins are αβγ hetero-trimers [43]. The α-subunits differ among the family members, and the β- and γ-subunits form a βγ dimer that serves as common scaffold for the binding of the α-subunit, to form specific oligomers [43]. In addition, pyruvate:ferredoxin oxidoreductase (POR) and 2-ketoisovalerate:ferredoxin oxidoreductase (VOR), from the hyperthermophilic archaeon *P*. *furiosus*, form αβγδ hetero-tetramers, which share the same γ-subunit but show different specificities for 2-oxoacid substrates, pyruvate (POR) and 2-ketoisovalerate (VOR) [44]. The three α, β and γ subunits are different between POR and VOR, and determine their substrate specificities. The POR and VOR genes are located in the single operon, porG(pf0971)-vorDAB(pf0972–0974)-porDAB(pf0975–0977) [44]. The subunit assembly strategies of GAOR2/3 and these protein complexes suggest that certain hetero-oligomeric proteins consist of a constant component “hostmer” (βγ-dimer of G-protein, α-subunit of hemoglobin, γ-subunit of POR/VOR and M/S-complex of GAOR2/3) and a variable component “guestmer” (α-subunit of G-protein, β/γ-subunit of hemoglobin, αβδ-subunit of POR/VOR and L-subunit of GAOR2/3) to achieve a variety of biological functions. Further studies, including the determination of the GAOR1 and GAOR3 structures, will be necessary to establish the proposed model on the subunit assembly in the GAORs.

In summary, we isolated and characterized the three GAORs from *S*. *tokodaii*, and found that GAOR2 and GAOR3 share the same M/S-subunits but contain different L-subunits, and exhibit distinct substrate specificities. We determined the crystal structure of GAOR2, as the first structure of an archaeal Mo-containing hydroxylase, thereby providing insights into the subunit assembly and the substrate specificity of the three GAORs. The structure of GAOR2 also enabled a comparative analysis of the Mo-hydroxylase enzymes from the three domains of life. Furthermore, our findings suggest that even primitive microbes are more complex than expected from their genomic information.

## Supporting Information

S1 Fig Modified Entner-Doudoroff (ED) glycolytic pathway of *S*. *tokodaii*. (PDF)

S2 Fig Isolation of the three GAORs. (PDF)

S3 Fig Absorption spectra of the GAORs. (PDF)

S4 Fig Electron density maps. (PDF)

S5 Fig Recognition of Mo-PCD (A), FAD (B) and PEG (C) (stereo views). (PDF)

S6 Fig Multiple amino-acid sequence alignment of the L-subunits. (PDF)

S7 Fig Substrate-binding sites (stereo views). (PDF)

S1 File Homology model, STK17810sm.pdb. (PDB)

S2 File Homology model, STK24840sm.pdb. (PDB)

S1 References (DOCX)

S1 Supporting Information (DOC)

S1 Table Inter-subunit Interactions in GAOR2. (DOCX)
