# Supplementary figures and images for: Archaeal Mo-Containing Glyceraldehyde Oxidoreductase Isozymes Exhibit Diverse Substrate Specificities through Unique Subunit Assemblies
Source: PLoS One. 2016 Jan 25;11(1):e0147333. doi: 10.1371/journal.pone.0147333 (PMC4726530; doi:10.1371/journal.pone.0147333)

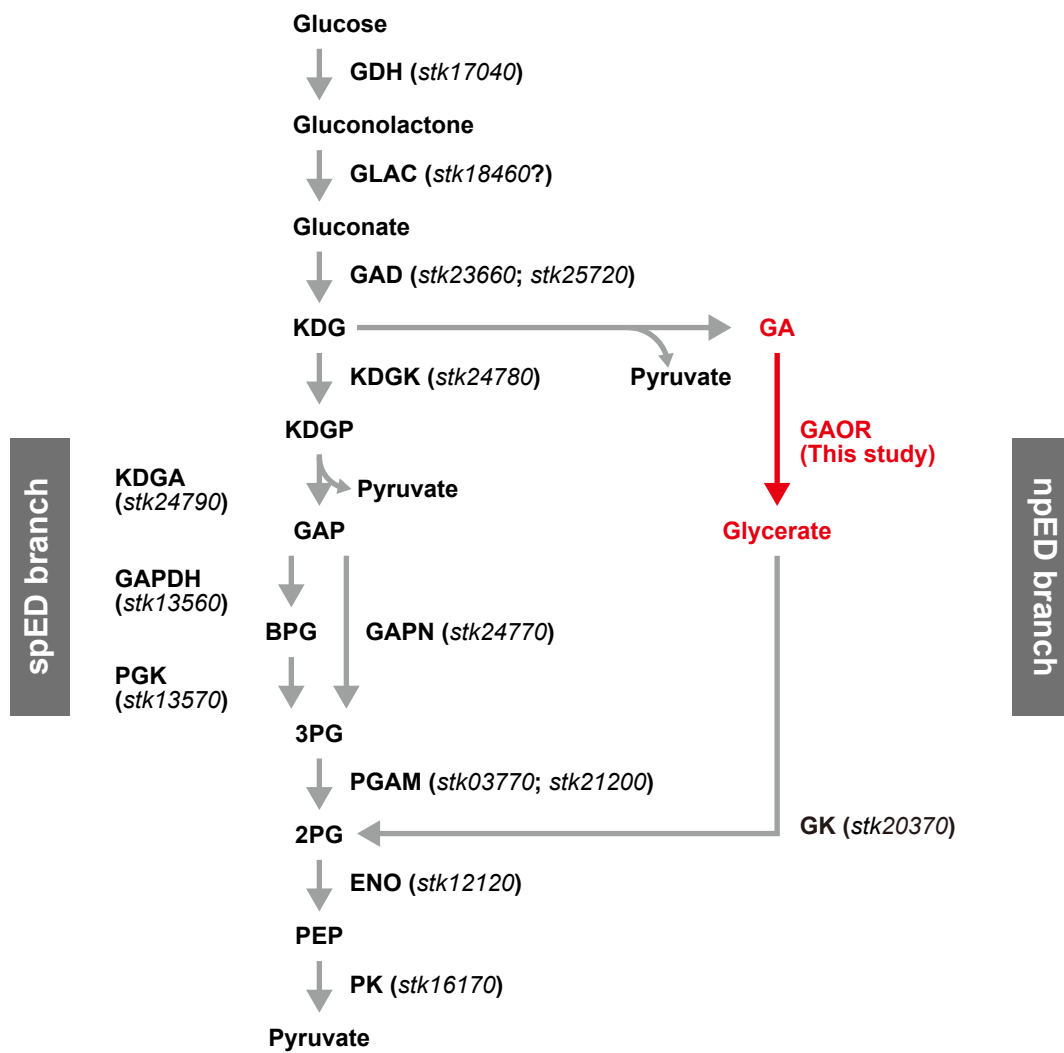

S1 Fig.

Supplement: S1 Fig — (PDF) [file pone.0147333.s001.pdf]

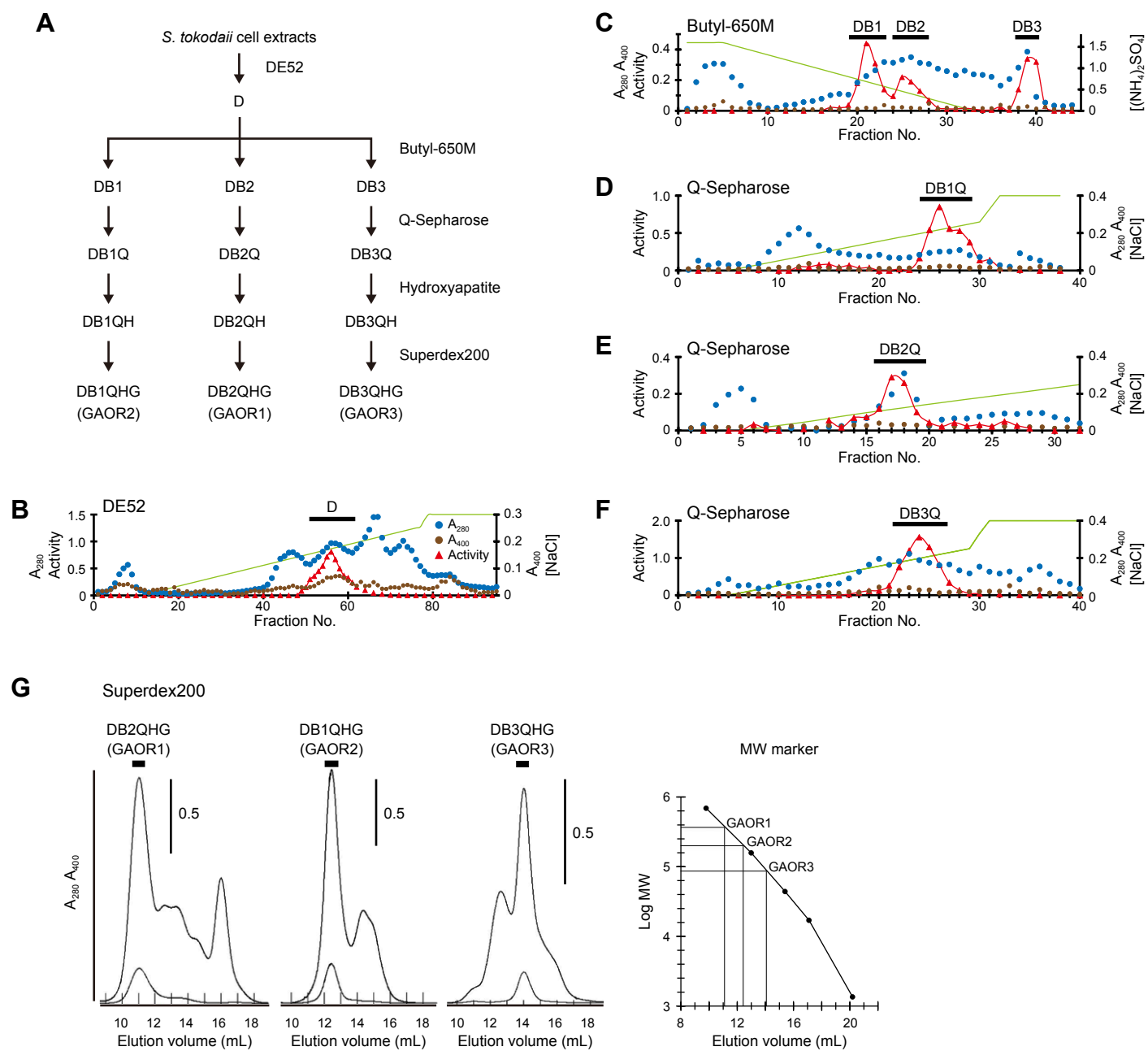

**S2 Fig.**

Supplement: S2 Fig — (PDF) [file pone.0147333.s002.pdf]

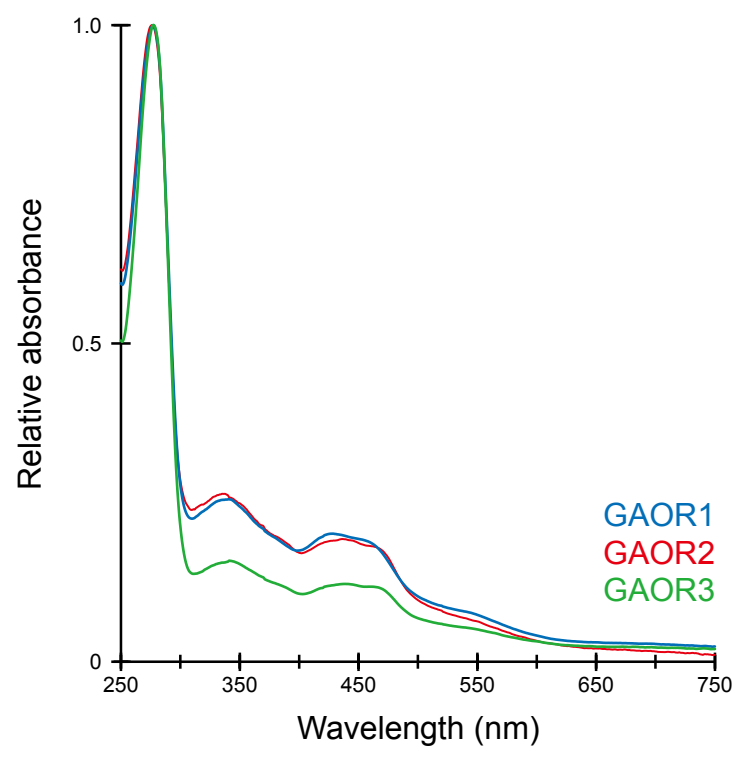

S3 Fig.

Supplement: S3 Fig — (PDF) [file pone.0147333.s003.pdf]

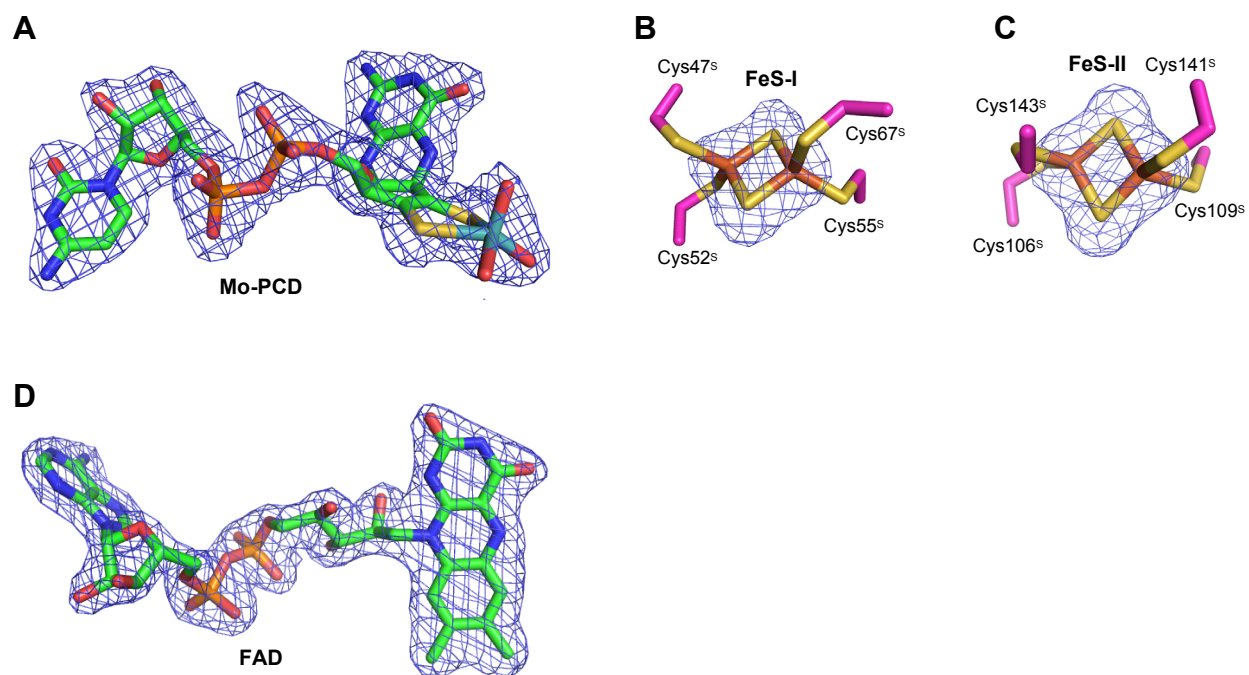

S4 Fig.

Supplement: S4 Fig — (PDF) [file pone.0147333.s004.pdf]

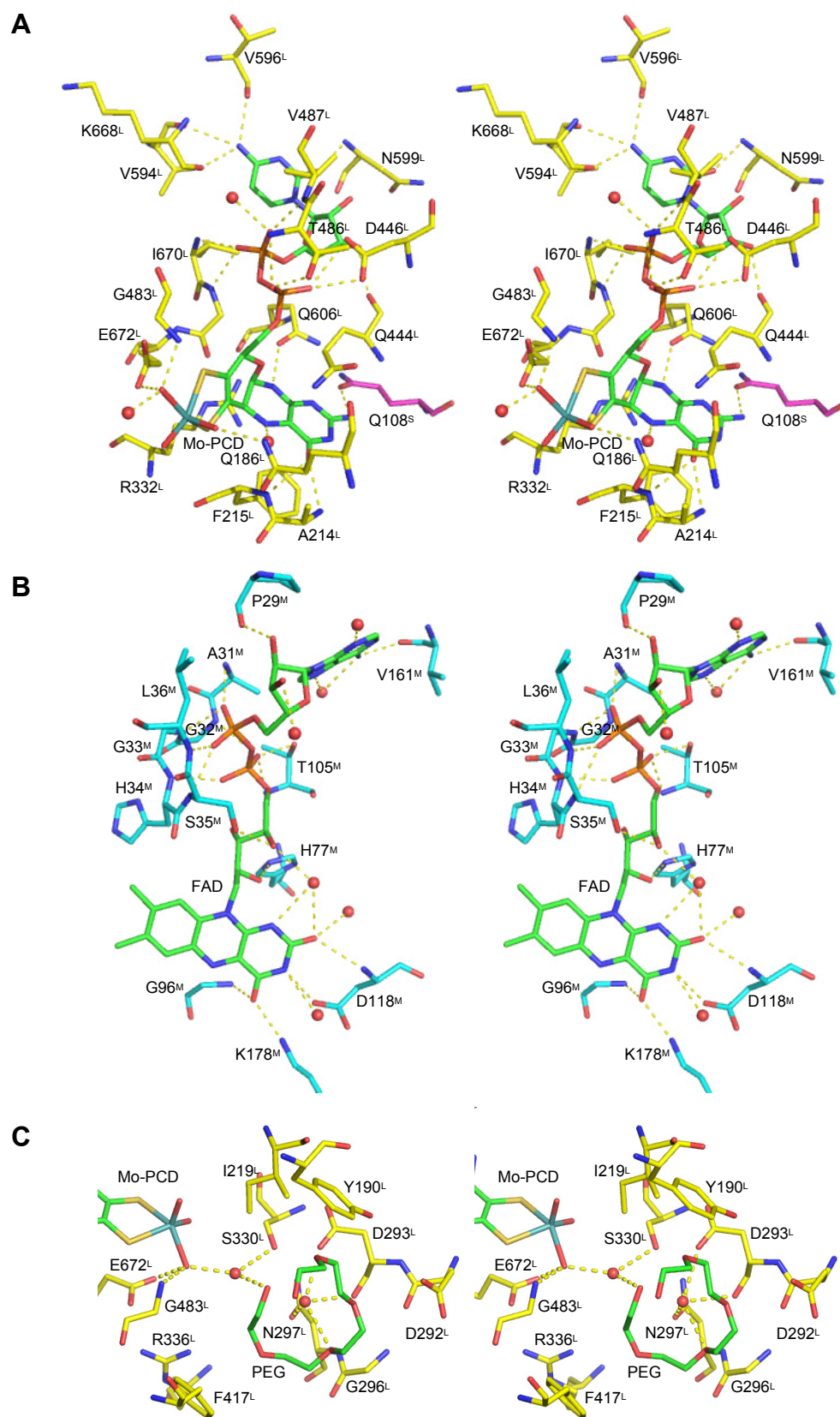

S5 Fig.

Supplement: S5 Fig — (PDF) [file pone.0147333.s005.pdf]

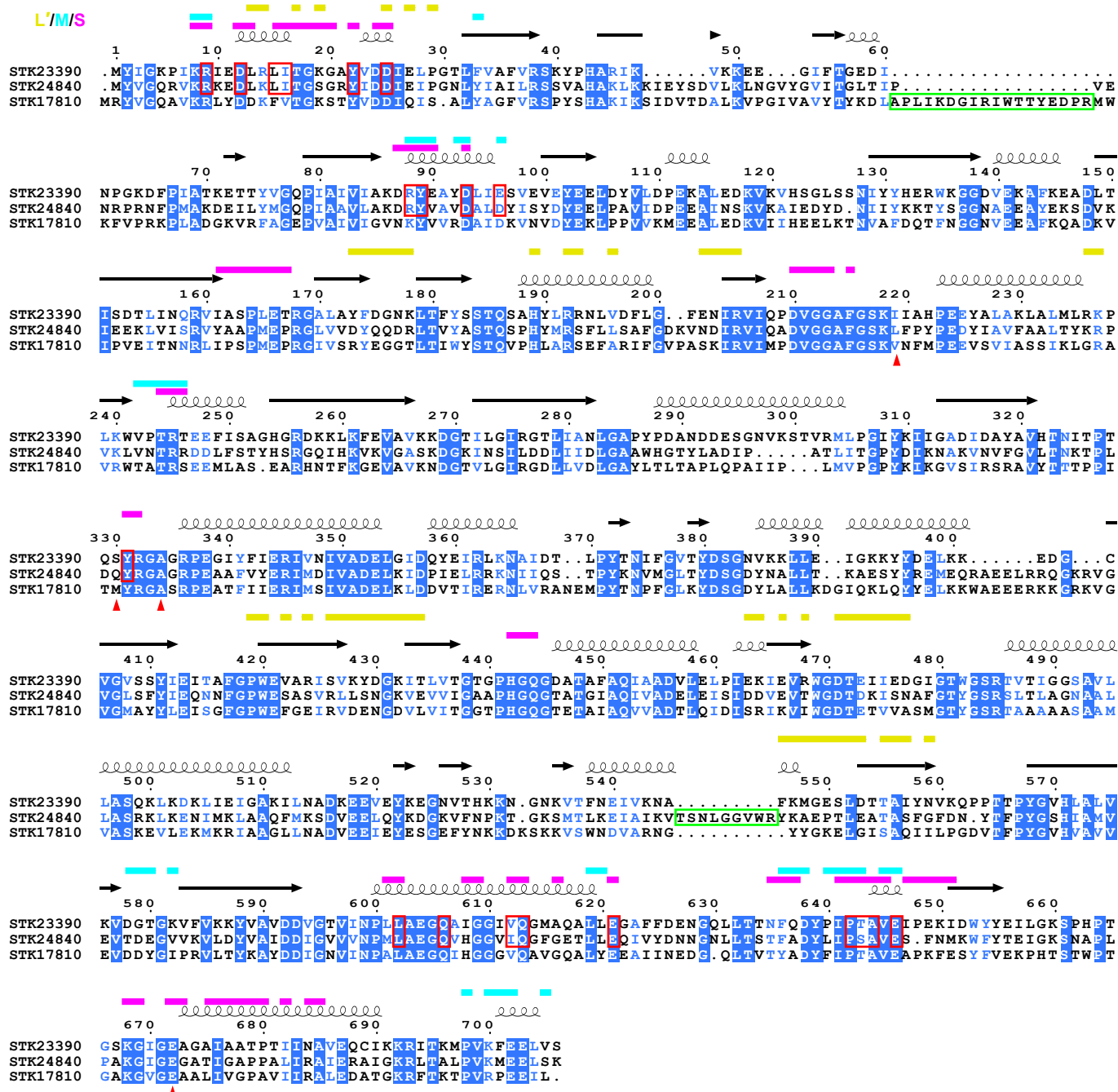

S6 Fig.

Supplement: S6 Fig — (PDF) [file pone.0147333.s006.pdf]

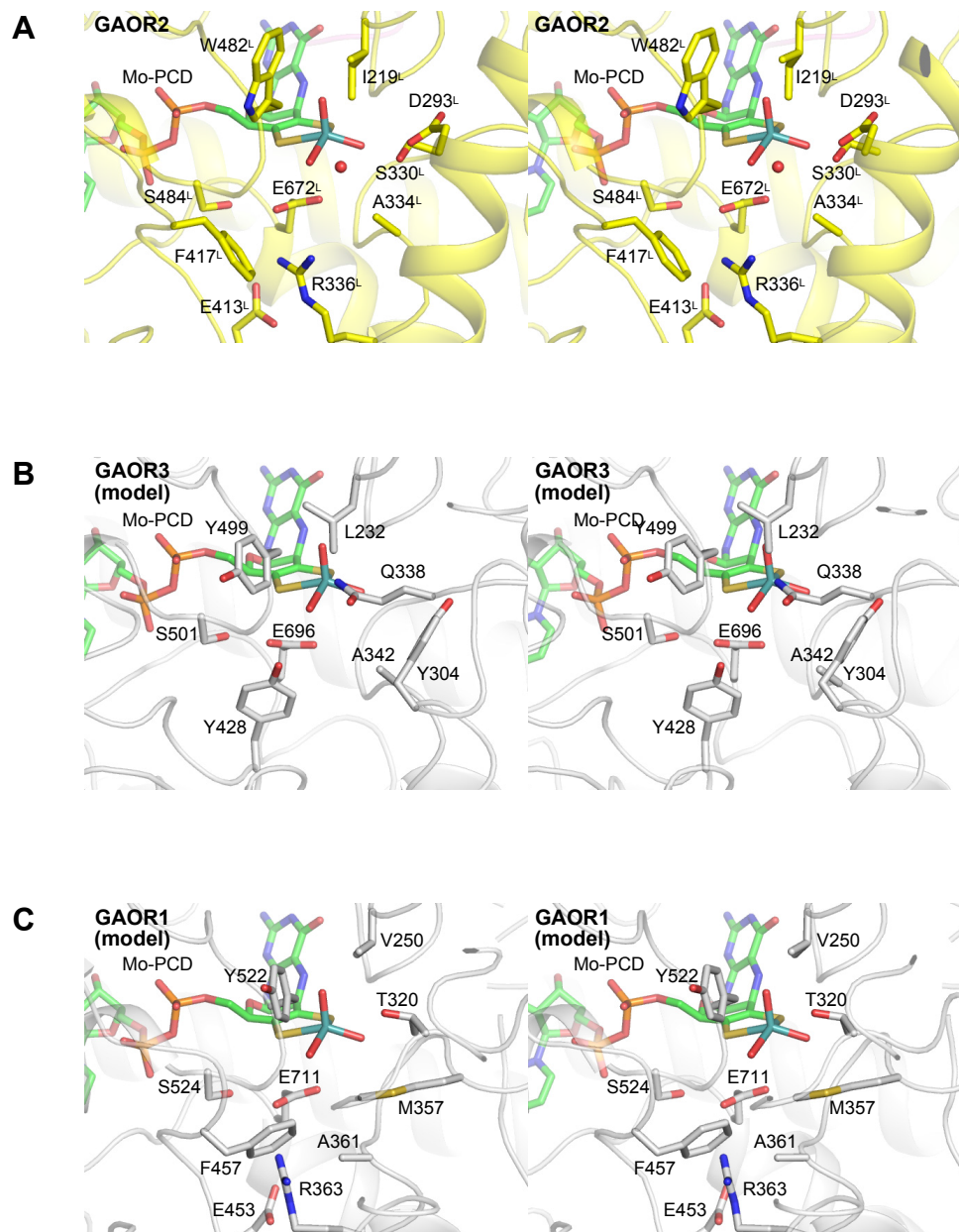

S7 Fig.

Supplement: S7 Fig — (PDF) [file pone.0147333.s007.pdf]
