# Supplementary material for: Archaeal Mo-Containing Glyceraldehyde Oxidoreductase Isozymes Exhibit Diverse Substrate Specificities through Unique Subunit Assemblies
Source: PLoS One. 2016 Jan 25;11(1):e0147333. doi: 10.1371/journal.pone.0147333 (PMC4726530; doi:10.1371/journal.pone.0147333)
Supplement: S1 References — (DOCX) [file pone.0147333.s010.docx]

**References**

1. Brasen C, Esser D, Rauch B, Siebers B (2014) Carbohydrate metabolism in Archaea: current insights into unusual enzymes and pathways and their regulation. Microbiol Mol Biol Rev. 78: 89-175.

2. Arnold K, Bordoli L, Kopp J, Schwede T (2006) The SWISS-MODEL workspace: a web-based environment for protein structure homology modelling, Bioinformatics. 22: 195-201.

3. Krissinel E, Henrick K (2007) Inference of macromolecular assemblies from crystalline state. J Mol Biol. 372: 774-797.
