# Supplementary material for: Archaeal Mo-Containing Glyceraldehyde Oxidoreductase Isozymes Exhibit Diverse Substrate Specificities through Unique Subunit Assemblies
Source: PLoS One. 2016 Jan 25;11(1):e0147333. doi: 10.1371/journal.pone.0147333 (PMC4726530; doi:10.1371/journal.pone.0147333)
Supplement: S1 Supporting Information — (DOC) [file pone.0147333.s011.doc]

**Supporting Information**

**S1 Fig. Modified Entner-Doudoroff (ED) glycolytic pathway of *S. tokodaii*.**

The nonphosphorylative ED (npED) and the semiphosphorylative ED (spED) are shown [1]. Abbreviations: BPG, 1,3-bisphosphoglycerate; ENO, enolase; GAD, gluconate dehydratase; GAOR, glyceraldehyde oxidoreductase; GAP, glyceraldehyde 3-phosphate; GAPDH, GAP dehydrogenase; GAPN, nonphosphorylating GAPDH; GDH, glucose dehydrogenase; GK, glycerate kinase; GLac, gluconolactonase; KDG, 2-keto-3-deoxygluconate; KDGK, KDG kinase; KDGA, KDG aldolase; KDPG, 2-keto-3-deoxy-6-phosphogluconate, PEP, phosphoenolpyruvate; 3PG, 3-phosphoglycerate; 2PG, 2-phosphoglycerate; PGK, phosphoglycerate kinase; PGL, 6-phosphoglucono-1,4-lactonase; PGAM, phosphoglycerate; PK, pyruvate kinase. The gene(s) corresponding to each enzyme are shown in parenthesis.

**S2 Fig. Isolation of the three GAORs.**

(**A**) Purification procedure of GAOR1–3 from *S. tokodaii* cell extracts.

(**B**–**F**) Elution profiles of the GAORs from DE52 (**B**), Butyl-650M (**C**), and Q-Sepharose (**D**–**F**) columns. A_280_, A_400_, and GA-oxidizing activities are indicated by blue circles, brown circles, and red triangles, respectively. The salt concentrations are indicated by green lines.

(**G**) Elution profiles of the GAORs from the Superdex200 gel filtration column. A_280_ and A_400_ are indicated by upper and lower lines, respectively. The column was calibrated with the standard molecular weight markers.

**S3 Fig. Absorption spectra of the GAORs.**

**Absorption spectra of GAORs.**

Absorption spectra of GAOR1 (0.76 mg/ml, A_280_ = 1.12), GAOR2 (0.39 mg/ml, A_280_ = 0.455), and GAOR3 (0.86 mg/ml, A_280_ = 1.10) in 50 mM Tris-HCl, pH 7.5 are indicated by blue, red, and green lines, respectively.

**S4 Fig. Electron density maps.**

|*F*_o_| – |*F*_c_| omit maps of MCD-Mo (**A**), [2Fe-2S] cluster I (**B**), [2Fe-2S] cluster II (**C**), and FAD (**D**) contoured at 3.5σ are shown as blue meshes.

**S5 Fig. Recognition of Mo-PCD (A), FAD (B) and PEG (C) (stereo views).**

Stereographic figures showing residues involved in binding of MCD-Mo (A), FAD (B) and PEG (C).

**S6 Fig. Multiple amino-acid sequence alignment of the L-subunits.**

The secondary structure of STK23390 (GAOR2) is indicated above the sequences. Residues involved in the subunit interactions with the dimer-related L- (designated as L’), M-, and S-subunits are indicated above the secondary structure marks by yellow, cyan and magenta lines, respectively. Key residues involved in the major interactions with the M/S-subunits (see Figs 3A-D) are boxed with red lines. The active site residues are indicated by red triangles. Insertions in STK17810 (GAOR1) and STK24840 (GAOR3) are boxed with green lines.

**S7 Fig. Substrate-binding sites (stereo views).**

(**A**–**C**): Crystal structure of STK23390 (GAOR2) (**A**) and the model structures of STK24840 (GAOR3) (**B**) and STK17810 (GAOR1) (**C**). Homology modeling was performed using the SWISS-MODEL server [2].

**S1 Table.** Inter-subunit interactions in GAOR2.

| Subunit pair | Interface area (Å^2^) | No. of hydrogen bonds | No. of salt bridges | No. of residues |
| --- | --- | --- | --- | --- |
| L–S | 2,122 | 33 | 12 | 62 (L), 56 (S) |
| L–L′ | 2,077 | 34 | 18 | 59 (L and L′) |
| M–S | 1,586 | 15 | 6 | 46 (M), 44 (S) |
| L–M | 930 | 12 | 9 | 32 (L), 24 (M) |

Calculated by the PDBePISA server [3].

**References**

1. Brasen C, Esser D, Rauch B, Siebers B (2014) Carbohydrate metabolism in Archaea: current insights into unusual enzymes and pathways and their regulation. Microbiol Mol Biol Rev. 78: 89-175.

2. Arnold K, Bordoli L, Kopp J, Schwede T (2006) The SWISS-MODEL workspace: a web-based environment for protein structure homology modelling, Bioinformatics. 22: 195-201.

3. Krissinel E, Henrick K (2007) Inference of macromolecular assemblies from crystalline state. J Mol Biol. 372: 774-797.

**Supporting files**

**S1 File. Homology model, STK17810sm.pdb**

Model information:

Modelled residue range: 4 to 744

Based on template: [1ffvB] (2.25 Å), B-chain of HETERO HEXAMER

Sequence Identity [%]: 31

Evalue: 0.00e-1

QMEAN Z-Score: -2.68

**S2 File. Homology Model, STK24840sm.pdb**

Model information:

Modelled residue range: 2 to 730

Based on template: [1rm6A] (1.60 Å) , A-chain of HETERO HEXAMER

Sequence Identity [%]: 27.06

Evalue: 0.00e-1

QMEAN Z-Score: -3.35
