# Supplementary material for: Archaeal Mo-Containing Glyceraldehyde Oxidoreductase Isozymes Exhibit Diverse Substrate Specificities through Unique Subunit Assemblies
Source: PLoS One. 2016 Jan 25;11(1):e0147333. doi: 10.1371/journal.pone.0147333 (PMC4726530; doi:10.1371/journal.pone.0147333)
Supplement: S1 Table — (DOCX) [file pone.0147333.s012.docx]

**S1 Table. Inter-subunit interactions in GAOR2.**

| Subunit pair | Interface area (Å^2^) | No. of hydrogen bonds | No. of salt bridges | No. of residues |
| --- | --- | --- | --- | --- |
| L–S | 2,122 | 33 | 12 | 62 (L), 56 (S) |
| L–L′ | 2,077 | 34 | 18 | 59 (L and L′) |
| M–S | 1,586 | 15 | 6 | 46 (M), 44 (S) |
| L–M | 930 | 12 | 9 | 32 (L), 24 (M) |

Calculated by the PDBePISA server [3].
